# Freeze-drying of “pearl milk tea”: A general strategy for controllable synthesis of porous materials

**DOI:** 10.1038/srep26438

**Published:** 2016-05-19

**Authors:** Yingke Zhou, Xiaohui Tian, Pengcheng Wang, Min Hu, Guodong Du

**Affiliations:** 1 The State Key Laboratory of Refractories and Metallurgy, College of Materials and Metallurgy, Wuhan University of Science and Technology, Wuhan, 430081, P. R. China

## Abstract

Porous materials have been widely used in many fields, but the large-scale synthesis of materials with controlled pore sizes, pore volumes, and wall thicknesses remains a considerable challenge. Thus, the controllable synthesis of porous materials is of key general importance. Herein, we demonstrate the “pearl milk tea” freeze-drying method to form porous materials with controllable pore characteristics, which is realized by rapidly freezing the uniformly distributed template-containing precursor solution, followed by freeze-drying and suitable calcination. This general and convenient method has been successfully applied to synthesize various porous phosphate and oxide materials using different templates. The method is promising for the development of tunable porous materials for numerous applications of energy, environment, and catalysis, etc.

Porous materials with unique porous structures and high surface areas have been widely used in many fields such as energy, catalysis, adsorption, separation, etc^1^,^2^,^3^,^4^,^5^,^6^,^7^,^8^,^9^. Currently, porous materials are typically obtained *via* template-assisted processes, during which the hard-templating (polymer- or silica-based colloidal crystals, anodic aluminum oxide and other porous solids) or soft-templating (surfactants, block copolymers) techniques are often used^5^,^10^,^11^,^12^. Although various advanced porous materials have been reported, the large-scale synthesis of materials with controlled pore sizes, pore volumes, and wall thicknesses remains a considerable challenge^5^,^13^. Therefore, synthesis strategies that enable the production of porous materials with controlled pore characteristics are of key general importance. Herein, we report a convenient “pearl milk tea” freeze-drying approach to synthesize porous materials with precisely tunable porous characteristics. The generality of our approach is demonstrated by describing the controllable synthesis of various porous phosphate, oxide, and composite materials.

The formation of porous materials by freeze-drying “pearl milk tea” is generally involved three steps, as schematically shown in Fig. 1. First, the raw materials and templates are dispersed and mixed into a small amount of solvent (water or some organic solvent) to form the precursor solution of “pearl milk tea”, where the template corresponds to the pearl. Second, the “pearl milk tea” is rapidly frozen and subsequently freeze-dried in vacuum to immobilize the template in the solid precursor and remove the solvent by sublimation. Third, the solid precursor is slowly calcined at a relatively low temperature to further condense the precursor, and it is subsequently annealed at a higher temperature to remove the templates and obtain the target porous materials. With this strategy, the pore size of the produced porous material is determined by the size of the applied template, whereas the pore distance and wall thickness are tuned using the ratio of template (namely, the concentration of template in the precursor). Thus, the porosity can be finely controlled. For widely used methods such as the colloidal crystal template process, the templates must be packed into a highly ordered film before infiltrating the precursor solution into the voids among the packed templates^5^,^12^. Therefore, although highly ordered reverse replica porous materials can be obtained using the colloidal crystal template method, the controllability of the pore distance, porosity and the large-scale production are partly restricted. The reason for this restriction is the constantly closest packing geometry of the colloidal template beads with a specific size^9^, as shown in Supplementary Figure S1. In contrast, in the current approach, the template beads (pearl) are initially freely dispersed in the precursor solution (milk tea) before they are suddenly frozen and solidified. Parameters such as the distance between the template beads and the template-to-precursor ratio can be easily controlled to finely and widely control the porous characteristics of the product.

## Results

Olivine lithium iron phosphate (LiFePO_4_) is an important positive electrode material of rechargeable lithium-ion batteries and is used in various applications such as portable electronic devices and electric vehicles^14^,^15^,^16^. Thus, LiFePO_4_ was selected as an example to demonstrate the effectiveness of this method. First, polymethyl methacrylate (PMMA), which has been widely used in the aforementioned colloidal crystal template process, was introduced. In a typical synthesis, PMMA templates with a uniform bead size were prepared by emulsion polymerization according to the literature^12^. Then, 4.04 g of iron nitrate, 1.02 g of lithium acetate and 0.68 mL of phosphoric acid were dissolved into a certain amount of distilled water, and the PMMA template was added. The mixture was stirred for 2 h to form the “pearl milk tea”, which was subsequently rapidly frozen using liquid nitrogen (−196 °C) and freeze-dried in a vacuum freezing dryer for 72 h. The solidified dry precursor was heated at 5 °C/min to 220 °C and maintained at this temperature for 3 h to further condense the inorganic precursor. Then, it was heated at the same rate to 600 °C and maintained for another 3 h in a reductive atmosphere of 90% argon and 10% hydrogen.

Figure 2a shows the SEM image of the PMMA template, which is a spherical particle with a uniform diameter of 100 nm. After they were well dispersed in the precursor solution, rapidly frozen and freeze-dried, the PMMA beads were uniformly mounted and immobilized in the solidified precursor, as shown in Fig. 2b. Upon the removal of the templates by calcination, the obtained materials replicated the size and morphology of the template and had a highly porous structure (Fig. 2c). The pores were notably uniform, and the pore size was approximately 100 nm, which is similar to the diameter of the applied template. X-ray diffraction studies indicate that the dried precursor remained amorphous and that the sample crystallized into a pure-phase olivine structure after annealing (Fig. 2d).

The porous characteristics of the olivine LiFePO_4_ material can be conventionally tuned by the experiment parameters. For example, the porosity, pore distance and wall thickness could be easily controlled by adjusting the template concentration (mass concentration of template to the target product, similarly hereinafter). Figure 3 displays the SEM images of the LiFePO_4_ products when the concentration of the applied template gradually increased from 25% to 80%. The pores were approximately 100 nm in size (similar to the diameter of the used templates) and were homogeneously distributed in these materials because of the uniform distribution of templates in the solidified precursor. When the template concentration increased from 25% to 80%, the surface area of the obtained materials remarkably increased from 18.59 to 94.58 m^2^/g, the porosity increased from 0.13 to 0.28 cm^3^/g (Supplementary Figure S2a), and the average pore wall thickness decreased from ~800 to 60 nm. The XRD patterns in Supplementary Figure S2b indicate that all of the materials crystallized into pure-phase olivine LiFePO_4_ (consistent with the standard diffraction card No: 01-083-2092). In addition, the PMMA templates with various bead sizes were synthesized by emulsion polymerization (30–200 nm, as shown in the SEM images in Supplementary Figure S3). When these templates were applied, porous LiFePO_4_ with various pore sizes of approximately 30, 50, 100, 130, 180 and 200 nm were obtained, as shown in Supplementary Figure S4, and the crystal structures were verified using XRD measurements (Supplementary Figure S5).

Importantly, this versatile method can be used to create porous structures in many different materials to tailor their physical, chemical, electrical and other properties. Similar to LiFePO_4_, porous olivine structural LiMPO_4_ (M = Mn, Co, and Ni), which is a promising positive electrode material with high potential and high theoretical energy density for lithium-ion batteries^17^,^18^, can be conveniently prepared using this “pearl milk tea” freeze-drying method by simply replacing iron nitrate with Mn, Co, or Ni containing nitrate. As shown in Supplementary Figure S6a–d, porous LiMnPO_4_, LiCoPO_4_, LiNiPO_4_, and LiFe_0.5_Mn_0.5_PO_4_ with pore diameters of approximately 100, 100, 150, and 100 nm, respectively, and an average wall thickness of 80–500 nm were easily prepared using PMMA templates with various diameters and concentrations (see the XRD results in Supplementary Figure S7). Such control of the electrode material is notably useful because the porous characteristics are relative to the electrode surface area, the electrode/electrolyte interface, the charge transfer ability and, consequently, the high-rate power capability^14^,^15^,^16^.

In addition to the phosphates, porous oxides can be effectively prepared using this synthesis strategy, as demonstrated by the successful synthesis of two important functional materials: TiO_2_ and Li_4_Ti_5_O_12_^1^,^19^,^20^,^21^. In this case, the choice of solvent is important because most soluble organic titanates (e.g., tetrabutyl titanate) and titanium-containing inorganic salts (e.g., TiCl_4_) tend to completely hydrolyze in water and form discrete nanoparticles. When tetrabutyl titanate is used as the source material and tertiary butyl alcohol, a widely used organic agent for the freeze-drying technique^22^, is used as the solvent, the frozen precursor melts again during the subsequent freeze-drying process, probably because of the exchange of butyl and tertiary butyl and the low freezing point of the produced butyl alcohol. Nevertheless, the use of titanium sulfate and tertiary butyl alcohol enables the successful synthesis of porous TiO_2_ and Li_4_Ti_5_O_12_ (with added Li salts) when using this method. As shown in Supplementary Figure S6e,f, uniformly distributed pores were created throughout both samples (see the XRD results in Supplementary Figure S8a,b). These results indicate that the “pearl milk tea” freeze-drying method can also be applied to organic-solvent systems.

To further investigate the generality of the “pearl milk tea” freeze-drying method, other widely used templates^23^ such as SiO_2_, polystyrene (PS), and polypyrrole (PPy) were applied and studied. Figure 4 displays typical SEM images of the applied templates, dry solid precursors, and calcined products of porous LiFePO_4_ using 100 nm PPy (Fig. 4a–c), porous MnO_2_ using 300 nm SiO_2_ (Fig. 4d–f), and porous MnO_2_ using 400 nm PS (Fig. 4g–i) (see the XRD results in Supplementary Figure S8c,d). Similar to PMMA, the PPy and PS templates were easily removed during the annealing process, but an additional etching process with HF solution after annealing was required when the SiO_2_ template was used because of its strong thermal stability. Similar phenomena and processes to the case of the PMMA template were clearly observed. These templates also helped produce homogeneously distributed porous materials, and the porous characteristics were highly related to the size and ratio of the applied templates.

## Discussion

**Figure 1:**
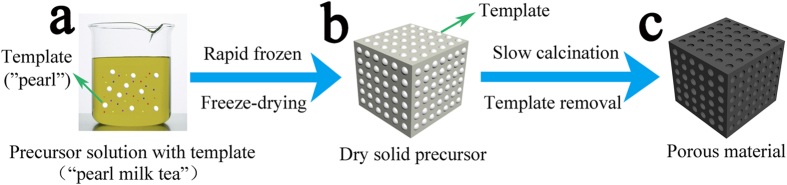
Schematic illustration of the formation process of porous materials using the “pearl milk tea” freeze-drying method. (**a**) The templates are dispersed in the precursor solution to form the “pearl milk tea”, and the templates correspond to the pearls; **(b**) Rapid freezing and freeze-drying to immobilize the templates and remove the solvent to obtain the dry solid precursor; (**c**) Slow calcination to remove the templates and obtain the porous material. The template beads (pearl) are initially freely dispersed in the precursor solution (milk tea) and suddenly frozen in liquid nitrogen. After freeze-drying, the templates remain highly dispersed in the dry solid precursor before removal with calcination. The porous characteristics of the product, such as the pore size, pore distance and porosity, can be widely controlled by tuning the template size and concentration.

**Figure 2:**
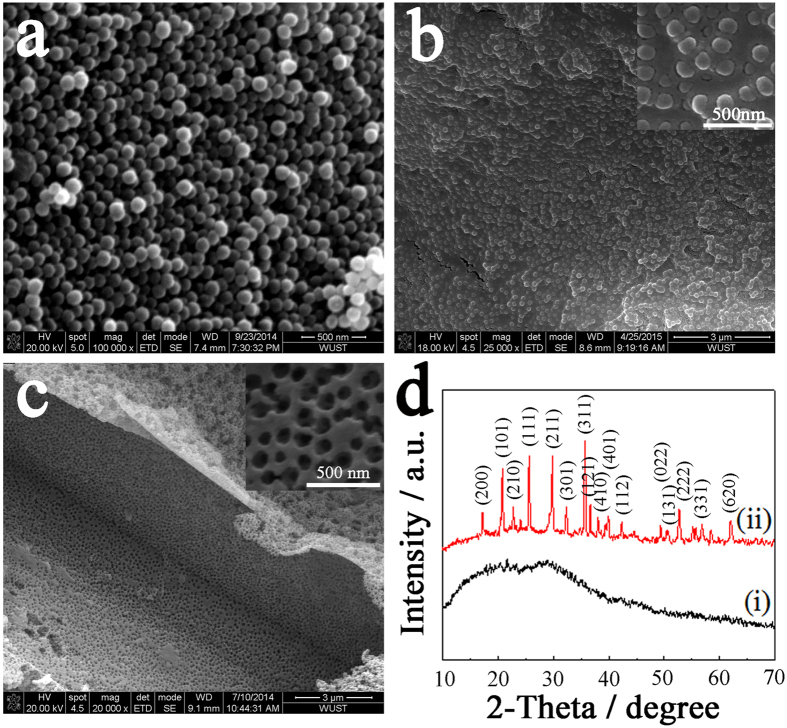
Morphology and structure evolution of the porous LiFePO_4_ material by the “pearl milk tea” freeze-drying method with the PMMA template. (**a**) SEM image of the PMMA template, which shows the uniform, smooth and spherical particles with a diameter of 100 nm. (**b**) SEM image of the dry solid precursor after the rapid freezing and freeze-drying of the precursor solution, which indicates that the templates are highly dispersed and uniformly embedded in the solid precursor. (**c**) SEM image of the porous material after the templates were removed by calcination; the obtained materials replicate the size and morphology of the template and have a highly porous structure with a pore diameter of approximately 100 nm. (**d**) XRD spectra of the dry solid precursor (i) and obtained porous material (ii), which indicate that the precursor remains amorphous, and the sample crystallizes into a pure-phase olivine structure after the calcination.

**Figure 3:**
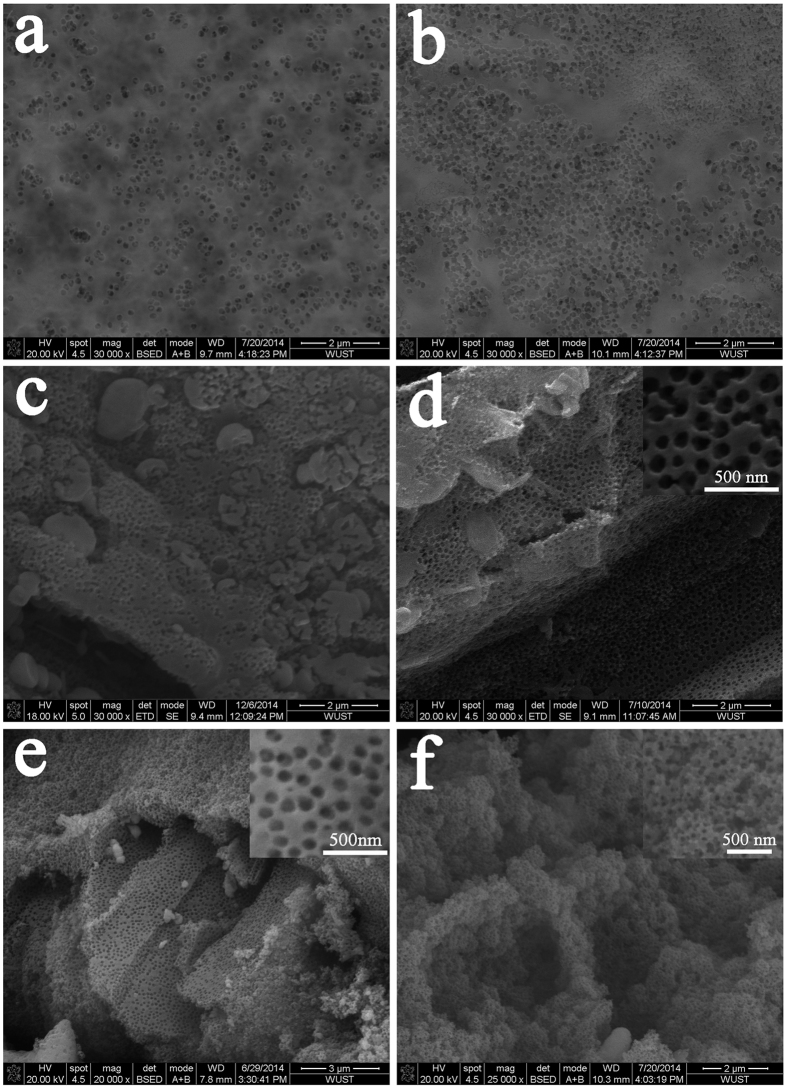
SEM images of porous LiFePO_4_ materials synthesized with PMMA templates of various concentrations. (**a**) 25 wt. %; **(b**) 33 wt. %; (**c**) 50 wt. %; (**d**) 67 wt. %; (**e**) 75 wt. %; (**f**) 80 wt. %. For all samples, the pores are approximately 100 nm in size, which is similar to the diameter of the applied template, and the average pore wall thickness decreases with the increase in template concentration.

**Figure 4:**
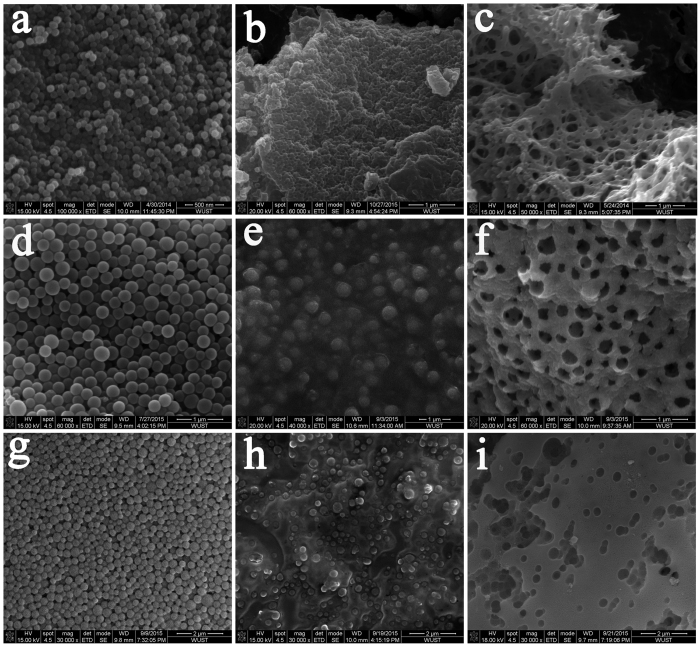
Morphology evolution of the formation process of porous materials synthesized by the “pearl milk tea” freeze-drying method with some other templates. (**a–c**) SEM images of the PPy template of 100 nm (**a**), dry solid precursor with the PPy template (**b**) and the produced porous LiFePO_4_ material (**c**). (**d**–**f**), SEM images of the SiO_2_ template of 300 nm (**d**), dry solid precursor with the SiO_2_ template (**e**) and the produced porous MnO_2_ material (**f**). (**g–i**) SEM images of the PS template of 400 nm (**g**), dry solid precursor with the PS template (**h**) and the produced porous MnO_2_ material (**i**). These results indicate that the “pearl milk tea” freeze-drying method can be applied to prepare different porous materials with various templates.

The microscopic reaction mechanisms of the “pearl milk tea” freeze-drying method are proposed as follows. Initially, both templates and ions (cation and anion) from the source materials are dissolved and homogeneously dispersed in a certain amount of solvent to form the sol-like tea (Fig. 1a). After their rapid freezing and subsequent freeze-drying in vacuum, the inorganic species may cross-link and form a continuous network among and surrounding the dispersive templates. For the process in an organic solvent, a small quantity of crosslinking agent (cellulose or PVP) is required to assist the oligomer and network formation; otherwise, only discrete nanoparticles are obtained. At this stage, no clear crystallization occurs, as verified by the XRD pattern of the almost amorphous structure (Fig. 2d(i)). When calcination is performed at a low temperature, the inorganic networks further condense to a firmer solid and subsequently crystallize into the final porous structure after the templates are removed by annealing at high temperature (however, the SiO_2_ template requires a further etching process).

The precise control of the porous structure (including the porosity, pore architecture, and pore size, etc.) in porous materials is significant for their properties and performance^24^. Currently, porous materials are mainly synthesized using soft-templating, hard-templating and non-templating methods^5^,^25^. The soft-templating approach typically uses a surfactant as the structure-directing agent, and the specific precursor and surfactant combinations must be appropriately selected. It is also difficult to obtain ordered and crystalline porous materials when the surfactant removal temperature is low because the crystallization of the inorganic phase at a higher temperature without support can destroy the desired porous structure^5^,^25^. For the hard-templating method, rigid structures such as anodic aluminum oxide membranes and silica- or polymer-based colloidal crystal films are pre-synthesized. Although highly crystallized and ordered porous materials can be obtained by using these templates, the wide controllability of pore characteristics and large-scale production are partly restricted. Compared to templating techniques, non-template methods such as sol-gel and hydrothermal syntheses are usually simpler in procedure. However, the porous architectures and ordering of the material are hardly controlled, and broader pore-size distributions are often obtained^5^,^25^. In comparison, the present “pearl milk tea” freeze-drying method combines the advantages of the templating synthesis and the freeze-drying technology, and highly crystallized pore architectures of materials can be effectively and precisely controlled. Moreover, the preparation of a highly ordered template framework similar to hard-templating is avoided, which simplifies the synthesis process and is suitable for the large-scale production of porous materials.

## Conclusion

In summary, we have demonstrated the “pearl milk tea” freeze-drying method to form porous materials with controllable pore characteristics such as the pore size, wall thickness, surface area and porosity. The unique strategy is realized by rapidly freezing the uniformly distributed template-containing precursor solution, followed by freeze-drying and suitable calcination. This method has been successfully applied to synthesize multi-element phosphates (LiFePO_4_, LiMPO_4_ (M = Mn, Co, Ni), and LiFe_0.5_Mn_0.5_PO_4_), multi-metal and single-metal oxides (Li_4_Ti_5_O_12_, TiO_2_, MnO_2_) porous materials with controllable pore characteristics using various templates (PMMA, PS, PPy, and SiO_2_) in either water or organic-solvent-based systems. Porous materials with even smaller pore sizes (e.g., sub-nano scale) may also be controllably synthesized using the current methodology, if the proper sub-nano template materials are selected and applied. Therefore, we believe that this general and convenient strategy can lead to rapid advancements in the development of various precisely tunable porous materials for numerous energy, environment, and catalysis applications.

## Methods

### Synthesis of PMMA template

The PMMA template was synthesized using an emulsion polymerization method according to the literature^12^. Sodium dodecyl sulfate (SDS) was used as the surfactant, and potassium peroxodisulfate (KPS) was used as the initiator. First, SDS was dissolved in distilled water at room temperature, and methyl methacrylate (MMA) was added before the solution was heated to the preset temperature. Then, the KPS solution was added drop-wise, and the reaction continued for 4 h at the same temperature. The solution began to turn opaque when the PMMA polymerization started; finally, a milky white colloidal solution formed. The templates were washed and collected by centrifugation and re-dispersed in the solvent. By carefully adjusting the synthesis conditions, a series of PMMA templates with various particle diameters (30–200 nm) were successfully synthesized, as summarized in Supplementary Table S1.

### Synthesis of PPy template

Polypyrrole (PPy) nanoparticles were synthesized in a water-soluble polymer/metal cation system according to a previous report^26^. Briefly, 2.58 g of polyvinyl alcohol (PVA) was dissolved in 204 mL of distilled water with stirring, and 7.46 g of FeCl_3_ was added. After a few minutes, pyrrole monomer was introduced, and the molar ratio of FeCl_3_ to pyrrole was set as 2.3. The solution turned black within a few minutes and was continuously stirred for 3 h. The PPy nanoparticles were washed several times with distilled water to remove the impurities and collected by suction filtration.

### Synthesis of PS template

The polystyrene (PS) template was synthesized by emulsion polymerization as described in the literature^27^. First, 5 mL of styrene was dissolved in 150 mL of distilled water. The solution was heated to 70 °C, and an aqueous solution of 0.25 M K_2_S_2_O_8_ was subsequently added to initialize the polymerization. The mixture was kept at 70 °C for 4 h, filtrated and washed to obtain the PS template.

### Synthesis of SiO_2_ template

The SiO_2_ template was synthesized according to the literature^28^. First, 9 mL of ammonia, 16 mL of ethanol and 25 mL of distilled water were mixed, and another solution of 4.5 mL tetraethyl orthosilicate (TEOS) and 45.5 mL ethanol was added. Then, the solution mixture was retained in an ice water bath for 6 h to obtain the suspension of SiO_2_ spheres. Finally, the products were centrifuged and washed with ethanol several times.

### Synthesis of porous LiFePO_4_

To prepare porous LiFePO_4_ samples, iron nitrate, lithium acetate, and phosphoric acid were used as the source materials. In a typical synthesis, 4.04 g of iron nitrate was first dissolved into 1.6 mL of distilled water; then, 1.02 g of lithium acetate and 0.68 mL of phosphoric acid were added, and the solution was stirred for 3 h to ensure homogeneity. Then, a specific amount of PMMA template was added, and the mixture was stirred for another 2 h. The mixture was subsequently rapidly frozen using liquid nitrogen and dried in a vacuum freezing dryer for 72 h. The dried samples were slowly heated to 220 °C, maintained at this temperature for 3 h to further condense the inorganic precursor, heated to 600 °C and maintained at this temperature for another 3 h. Various amounts of PMMA templates and templates with different diameters were used to tune the porous characteristics of the product. The samples were calcined in a reductive atmosphere (90% argon and 10% hydrogen) to ensure that Fe (III) was completely reduced to Fe (II) species. In addition, the porous LiFePO_4_ sample was prepared with the PPy template. The synthesis processes were similar to those with PMMA, but the PPy template was used.

### Synthesis of porous LiMPO_4_ (M = Mn, Co, Ni) and LiMn_0.5_Fe_0.5_PO_4_

The synthesis processes of porous LiMPO_4_ (M = Mn, Co, Ni) and LiMn_0.5_Fe_0.5_PO_4_ were identical to those of LiFePO_4_, except Fe(NO_3_)_3_·9H_2_O was replaced by Mn(NO_3_)_2_·4H_2_O, Co(NO_3_)_2_·6H_2_O, Ni(NO_3_)_2_·6H_2_O or their combination.

### Synthesis of porous Li_4_Ti_5_O_12_ and TiO_2_

First, 0.2 g of ethyl cellulose was dissolved into 20 mL of tertiary butyl alcohol, and 6.0 g of Ti(SO_4_)_2_ and 2.1 g of LiH_2_PO_4_ were added. Then, the solution was heated to 50 °C and stirred for 3 h to ensure the homogeneity. Subsequently, 2.3 g of PMMA template was added to this solution, and the mixture was stirred for another 2 h. The mixture was rapidly frozen using liquid nitrogen and dried in a vacuum freezing dryer for 72 h. Then, the sample was heated at 5 °C /min to 220 °C and held at this temperature for 3 h to further condense the inorganic precursor. Subsequently, the sample was heated at the same rate to 800 °C and remained at this temperature for 16 h. The final product was obtained after natural cooling to room temperature. For porous TiO_2_, the synthesis process was similar to that of Li_4_Ti_5_O_12_, except for the absence of LiH_2_PO_4_.

### Synthesis of porous MnO_2_

Porous MnO_2_ materials were synthesized with both PS and SiO_2_ templates. For the PS template, typically, 0.20 g of PVP and 12.55 g of Mn(NO_3_)_2_·4H_2_O were first dissolved in 10 mL tertiary butyl alcohol, and 1.45 g of PS was added. The mixture was stirred for 4 h, rapidly frozen using liquid nitrogen, and dried in the vacuum freezing dryer for 72 h. Finally, porous MnO_2_ was obtained after calcination in air at 400 °C for 3 h. For the SiO_2_ template, all procedures were similar, except the SiO_2_ template was used instead of PS. Because of the thermal stability of SiO_2_, a diluted hydrofluoric acid was used to etch away the templates after the thermal treatments. Porous MnO_2_ was finally obtained after washing with distilled water and drying in a vacuum drying oven.

### Characterization

X-ray powder diffraction (XRD) was used to identify the purity and crystallinity of the samples, and the diffraction data were collected using an Xpert Pro MPD diffractometer with Cu K_α_ radiation (*λ* = 0.15418 nm) and 2*θ* angles from 20° to 80°. SEM investigations were conducted using a scanning electron microscope (SEM, PHILIPS XL30 TMP) with an acceleration voltage of 15 kV. The nitrogen sorption isotherms were measured on Autosorb-1-MP/LP at the boiling point of liquid nitrogen, and the specific surface area was calculated using the multipoint Brunauer-Emmett-Teller (BET) method.

## Additional Information

**How to cite this article**: Zhou, Y. *et al.* Freeze-drying of “pearl milk tea”: A general strategy for controllable synthesis of porous materials. *Sci. Rep.* **6**, 26438; doi: 10.1038/srep26438 (2016).

## Supplementary Material

Supplementary Information

## References

[b1] Crossland E. J. *et al.* Mesoporous TiO_2_ single crystals delivering enhanced mobility and optoelectronic device performance. Nature 495, 215–219 (2013). 23467091 10.1038/nature11936

[b2] Chen Q. & Sieradzki K. Spontaneous evolution of bicontinuous nanostructures in dealloyed Li-based systems. Nature Mater. 12, 1102–1106 (2013). 23975058 10.1038/nmat3741

[b3] Zhou D., Cui Y., Xiao P., Jiang M. & Han B. A general and scalable synthesis approach to porous graphene. Nature Commun. 5, 4716 (2014). 25178835 10.1038/ncomms5716

[b4] Sun X. *et al.* Container effect in nanocasting synthesis of mesoporous metal oxides. J. Am. Chem. Soc. 133, 14542–14545 (2011). 21861449 10.1021/ja2060512

[b5] Vu A., Qian Y. & Stein A. Porous electrode materials for lithium-ion batteries - how to prepare them and what makes them special. Adv. Energy Mater. 2, 1056–1085 (2012).

[b6] Li W., Yue Q., Deng Y. & Zhao D. Ordered mesoporous materials based on interfacial assembly and engineering. Adv. Mater. 25, 5129–5152 (2013). 23868196 10.1002/adma.201302184

[b7] Dutta S., Wu K. C. & Kimura T. Predictable shrinkage during the precise design of porous materials and nanomaterials. Chem. Mater. 27, 6918–6928 (2015).

[b8] Huck J. M. *et al.* Evaluating different classes of porous materials for carbon capture. Energy Environ. Sci. 7, 4132–4146 (2014).

[b9] Petkovich N. D. & Stein A. Controlling macro- and mesostructures with hierarchical porosity through combined hard and soft templating. Chem. Soc. Rev. 42, 3721–3739 (2013). 23072972 10.1039/c2cs35308c

[b10] Zhuo S., Zhang J., Shi Y., Huang Y. & Zhang B. Self-template-directed synthesis of porous perovskite nanowires at room temperature for high-performance visible-light photodetectors. Angew. Chem. Int. Ed. 54, 5693–5696 (2015). 10.1002/anie.201411956 25776103

[b11] Vu A. *et al.* Three-dimensionally ordered mesoporous (3DOM) carbon materials as electrodes for electrochemical double-layer capacitors with ionic liquid electrolytes. Chem. Mater. 25, 4137–4148 (2013).

[b12] Doherty C. M., Caruso R. A., Smarsly B. M. & Drummond C. J. Colloidal crystal templating to produce hierarchically porous LiFePO_4_ electrode materials for high power lithium ion batteries. Chem. Mater. 21, 2895–2903 (2009).

[b13] Qian L. *et al.* Systematic tuning of pore morphologies and pore volumes in macroporous materials by freezing. J. Mater. Chem. 19, 5212–5219 (2009).

[b14] Gibot P. *et al.* Room-temperature single-phase Li insertion/extraction in nanoscale Li_x_FePO_4_. Nature Mater. 7, 741–747 (2008). 18660813 10.1038/nmat2245

[b15] Zhou Y. K., Wang J., Hu Y. Y., O’Hayre R. & Shao Z. P. A porous LiFePO_4_ and carbon nanotube composite. Chem. Commun. 46, 7151–7153 (2010). 10.1039/c0cc01721c 20676441

[b16] Ohmer N. *et al.* Phase evolution in single-crystalline LiFePO_4_ followed by *in situ* scanning X-ray microscopy of a micrometre-sized battery. Nature Commun. 6, 6045 (2015). 25599854 10.1038/ncomms7045

[b17] Hu M., Pang X. & Zhou Z. Recent progress in high-voltage lithium ion batteries. J. Power Sources 237, 229–242 (2013).

[b18] Julien C. M. & Mauger A. Review of 5-V electrodes for Li-ion batteries: status and trends. Ionics 19, 951–988 (2013).

[b19] Chen C. *et al.* Na^+^ intercalation pseudocapacitance in graphene-coupled titanium oxide enabling ultra-fast sodium storage and long-term cycling. Nature Commun. 6, 6929 (2015). 25906991 10.1038/ncomms7929

[b20] Wang J., Zhou Y. K. & Shao Z. P. Porous TiO_2_(B)/anatase microspheres with hierarchical nano and microstructures for high-performance lithium-ion batteries. Eletrochim. Acta 97, 386–392 (2013).

[b21] Sun Y. *et al.* Direct atomic-scale confirmation of three-phase storage mechanism in Li_4_Ti_5_O_12_ anodes for room-temperature sodium-ion batteries. Nature Commun. 4, 1870 (2013). 23695664 10.1038/ncomms2878

[b22] Qian L. & Zhang H. Controlled freezing and freeze drying: a versatile route for porous and micro-/nano-structured materials. J. Chem. Technol. Biotechnol. 86, 172–184 (2011).

[b23] Stein A., Rudisill S. G. & Petkovich N. D. Perspective on the influence of interactions between hard and soft templates and precursors on morphology of hierarchically structured porous materials. Chem. Mater. 26, 259–276 (2014).

[b24] Yu F. *et al.* Three-dimensional porous LiFePO_4_: Design, architectures and high performance for lithium ion batteries. Curr. Inorg. Chem. 2, 194–212 (2012).

[b25] Linares N., Silvestre-Albero A. M., Serrano E., Silvestre-Albero J. & García-Martínez J. Mesoporous materials for clean energy technologies. Chem. Soc. Rev. 43, 7681–7717 (2014). 24699503 10.1039/c3cs60435g

[b26] Hong J., Jeon S. O., Jang J., Song K. & Kim S. H. A facile route for the preparation of organic bistable memory devices based on size-controlled conducting polypyrrole nanoparticles. Org. Electron. 14, 979–983 (2013).

[b27] Yim C. H., Baranova E. A., Abu-Lebdeh Y. & Davidson I. Highly ordered LiFePO_4_ cathode material for Li-ion batteries templated by surfactant-modified polystyrene colloidal crystals. J. Power Sources 205, 414–419 (2012).

[b28] Abraham S. *et al.* Enhanced electrochemical biosensing efficiency of silica particles supported on partially reduced graphene oxide for sensitive detection of cholesterol. J. Electroanal. Chem. 757, 65–72 (2015).

