# Supplementary material for: Freeze-drying of “pearl milk tea”: A general strategy for controllable synthesis of porous materials
Source: Sci Rep. 2016 May 19;6:26438. doi: 10.1038/srep26438 (PMC4872234; doi:10.1038/srep26438)
Supplement: Supplementary Information [file srep26438-s1.doc]

**Supplementary information**

**Freeze drying of “pearl milk tea”: A general strategy for controllable synthesis of porous materials**

**Yingke Zhou**[[1]](#footnote-2)***, Xiaohui Tian, Pengcheng Wang, Min Hu, Guodong Dou**

The State Key Laboratory of Refractories and Metallurgy, College of Materials and Metallurgy, Wuhan University of Science and Technology, Wuhan, 430081, P. R. China.

^*^[zhouyk888@hotmail.com](mailto:zhouyk888@hotmail.com)


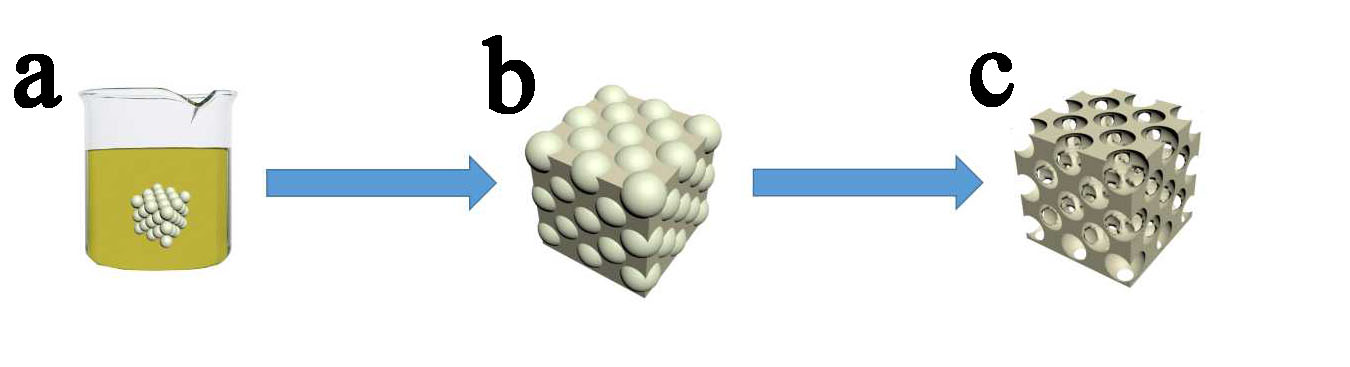


Porous material with interconnected pore structure

Dry colloidal crystal template containing precursor

Infiltration of colloidal crystal templates with precursor solution

Drying

Calcination

Template removal

**Figure S1. Schematic illustration of the preparation of porous material by the conventional colloidal crystal template method.** (**a**) Infiltration of precursor solution into the voids between the closely packed colloidal crystal templates. (**b**) Drying to obtain the dry colloidal crystal templates containing precursor. (**c**) Calcination to remove the colloidal crystal templates and get the porous materials with interconnected pore structure. Before infiltration the templates are required to pack into a highly ordered film, and then the voids between the packed templates are used to deposit the precursor of target material. Because of the constantly closest packing geometry form of the colloidal template beads with some specific size, interconnected porous structures are usually obtained, and the pore distance, porosity and the large scale production are partly restricted.

**Figure S2. Structural properties of the porous LiFePO_4_ materials synthesized with PMMA templates of various concentrations.** (**a**) Surface area and porosity of the obtained porous materials as a function of the template concentration, indicating that both surface area and porosity increase greatly with the concentration increase of PMMA template. (**b**) XRD patterns of porous materials synthesized with PMMA template concentrations of 25 wt.% (**i**), 33 wt.% (**ii**), 50 wt.% (**iii**), 67 wt.% (**iv**) and 80 wt.% (**v**). All the materials can be indexed on the basis of a pure phase orthorhombic olivine structure with a Pnma space group (JCPDS card No. 83-2092).


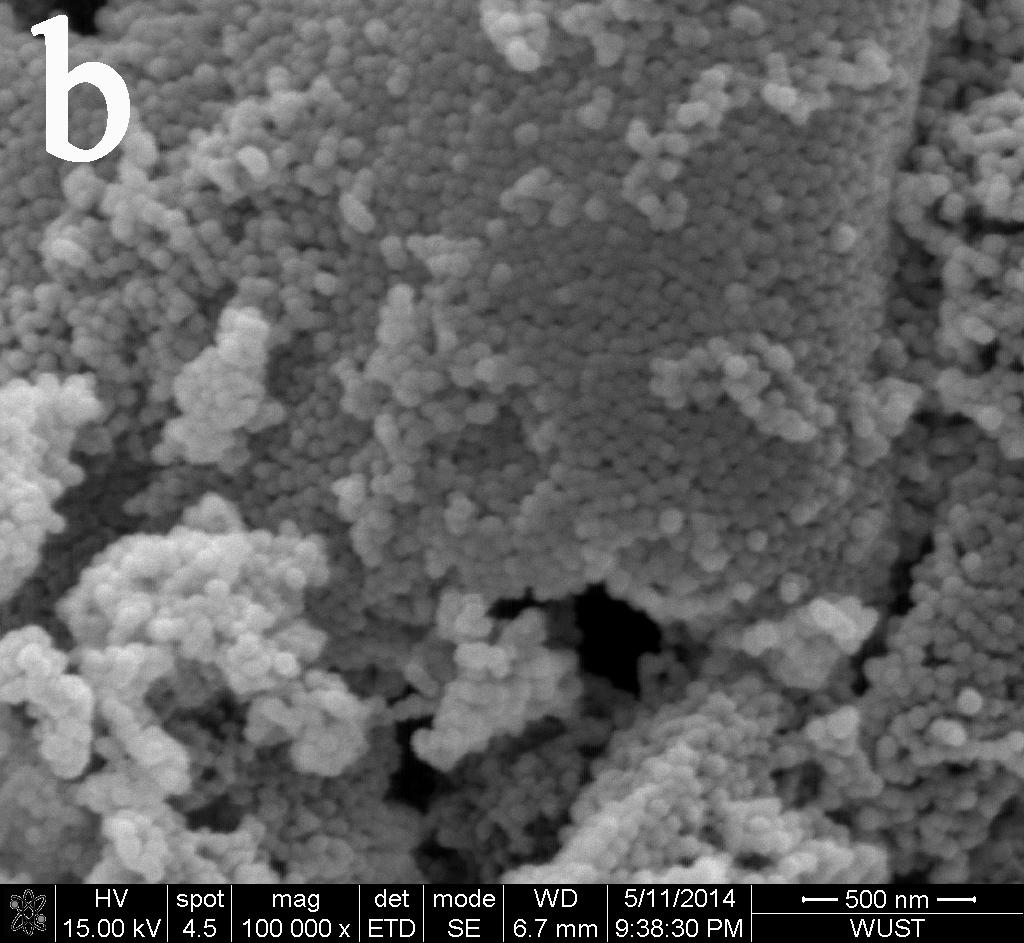

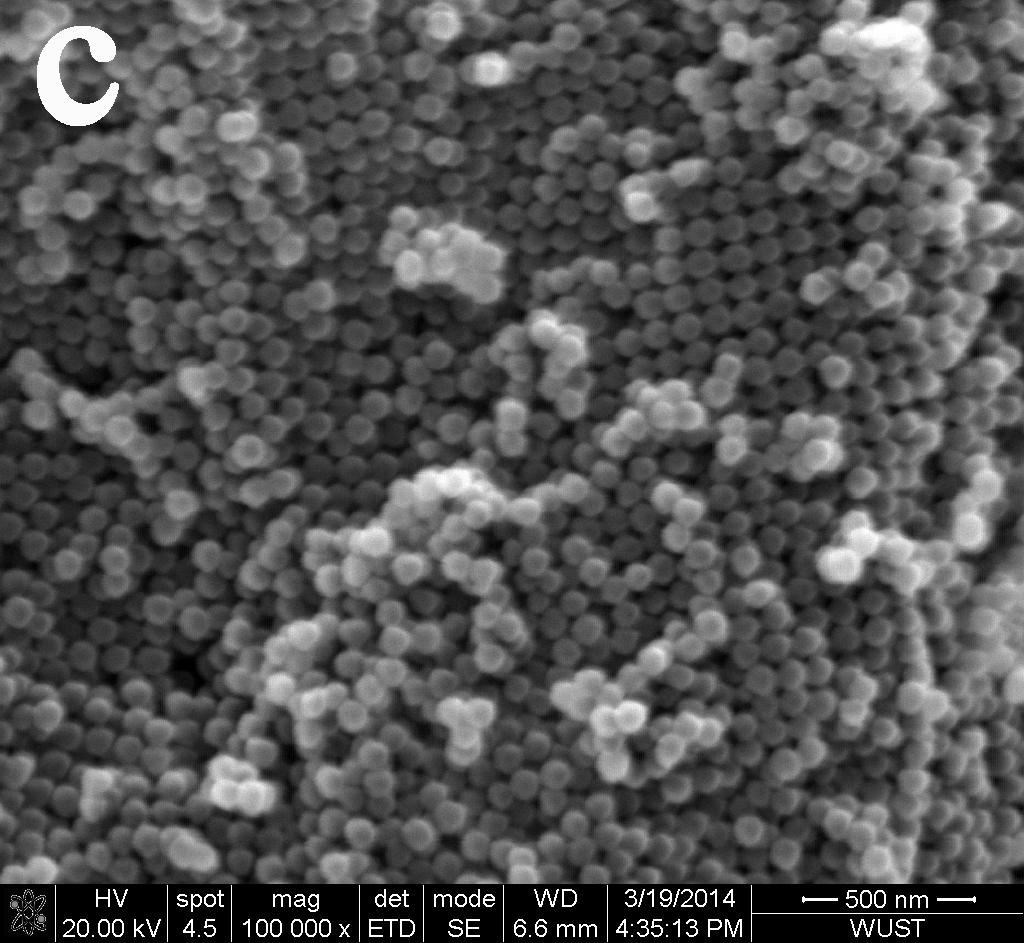

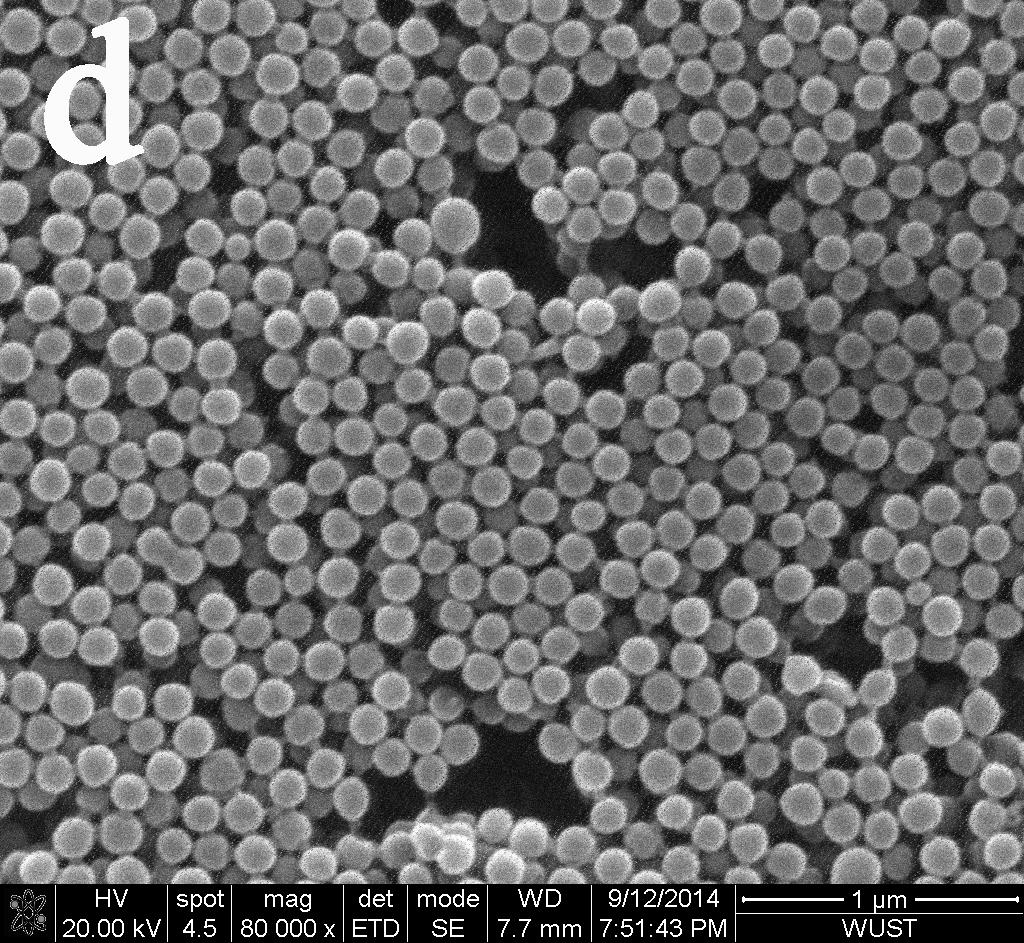

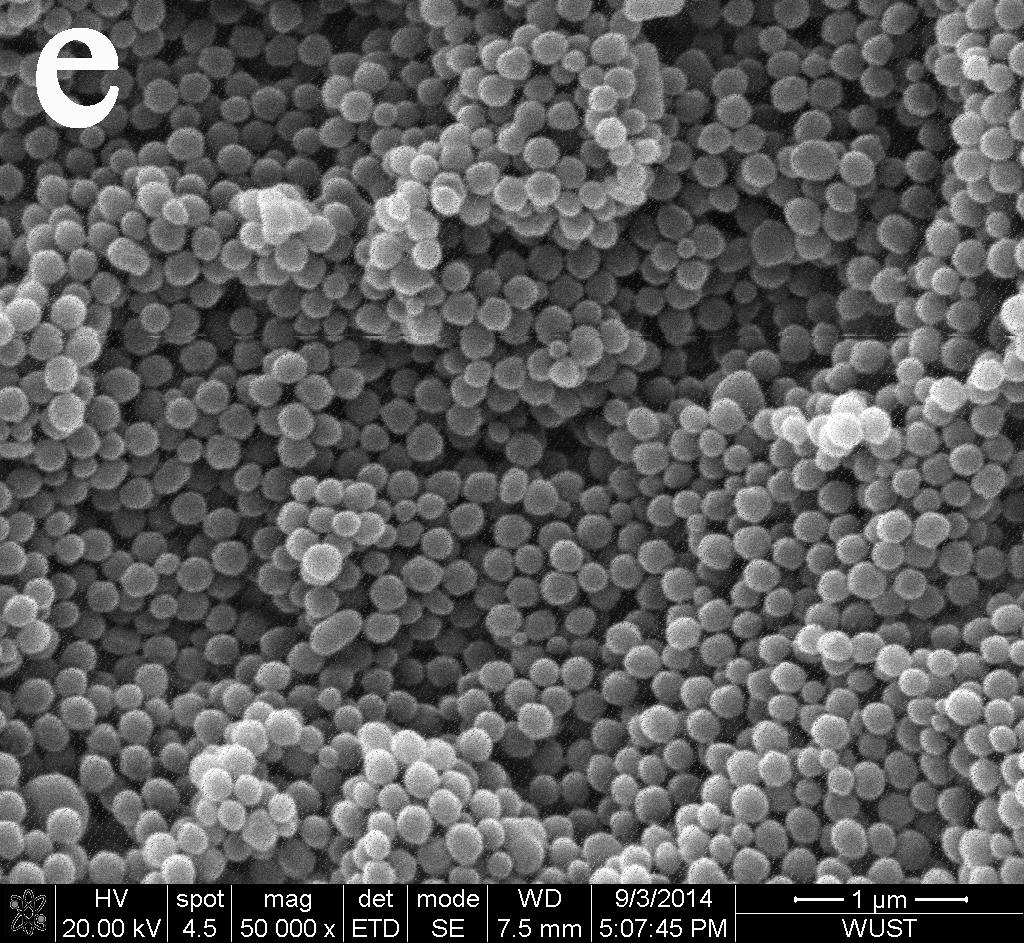

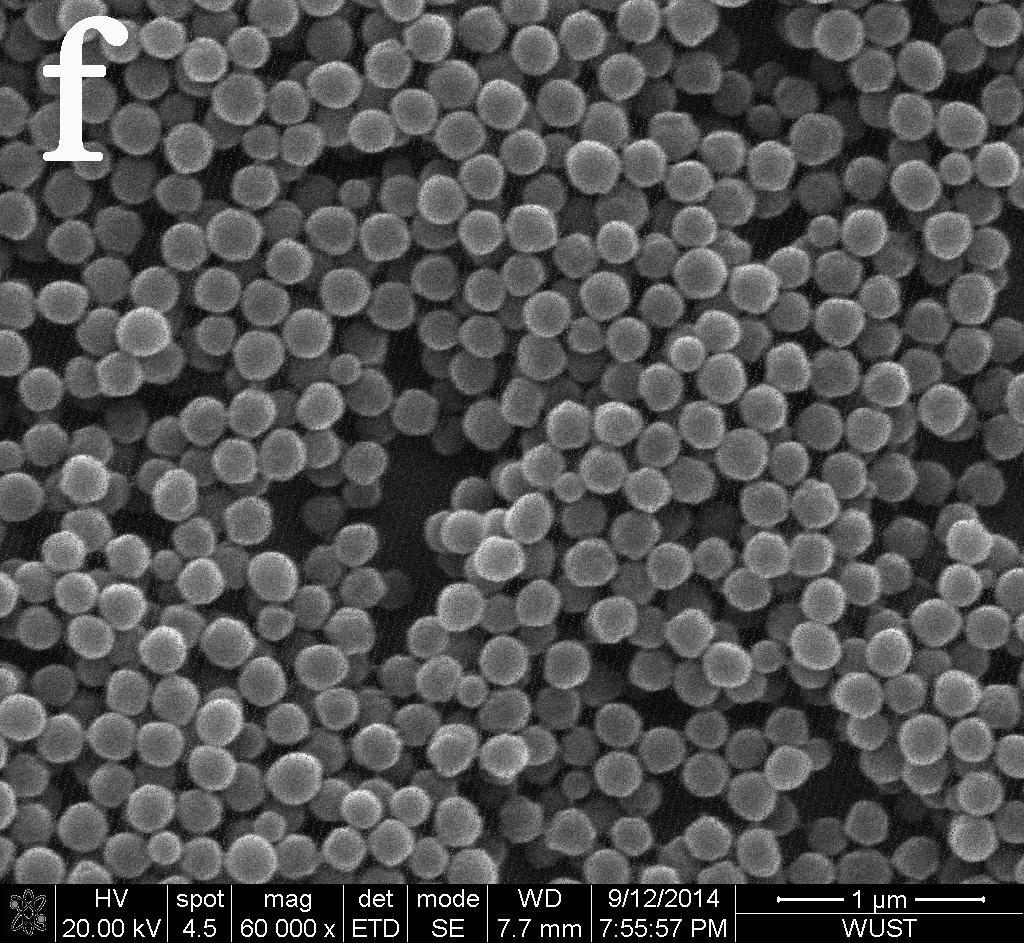

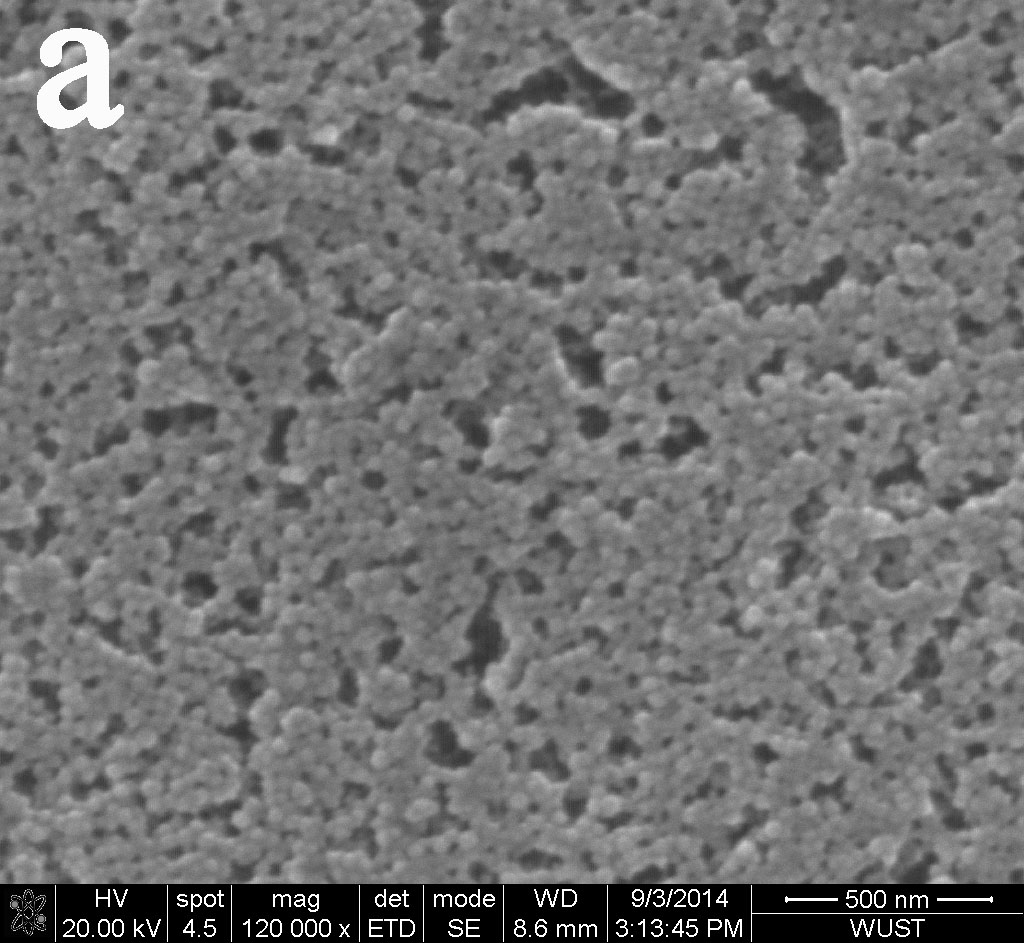


2

**Figure S3. SEM images of PMMA templates with various particle diameters.** (**a**) 30 nm; (**b**) 50 nm; (**c)** 100 nm; (**d**) 130 nm; (**e**) 180 nm; (**f**) 200 nm. These results show that the template particles are spherical and uniform, and the particle size can be finely controlled, which is beneficial to tune the pore size of the templated porous structures by using the “pearl milk tea” freeze drying method.


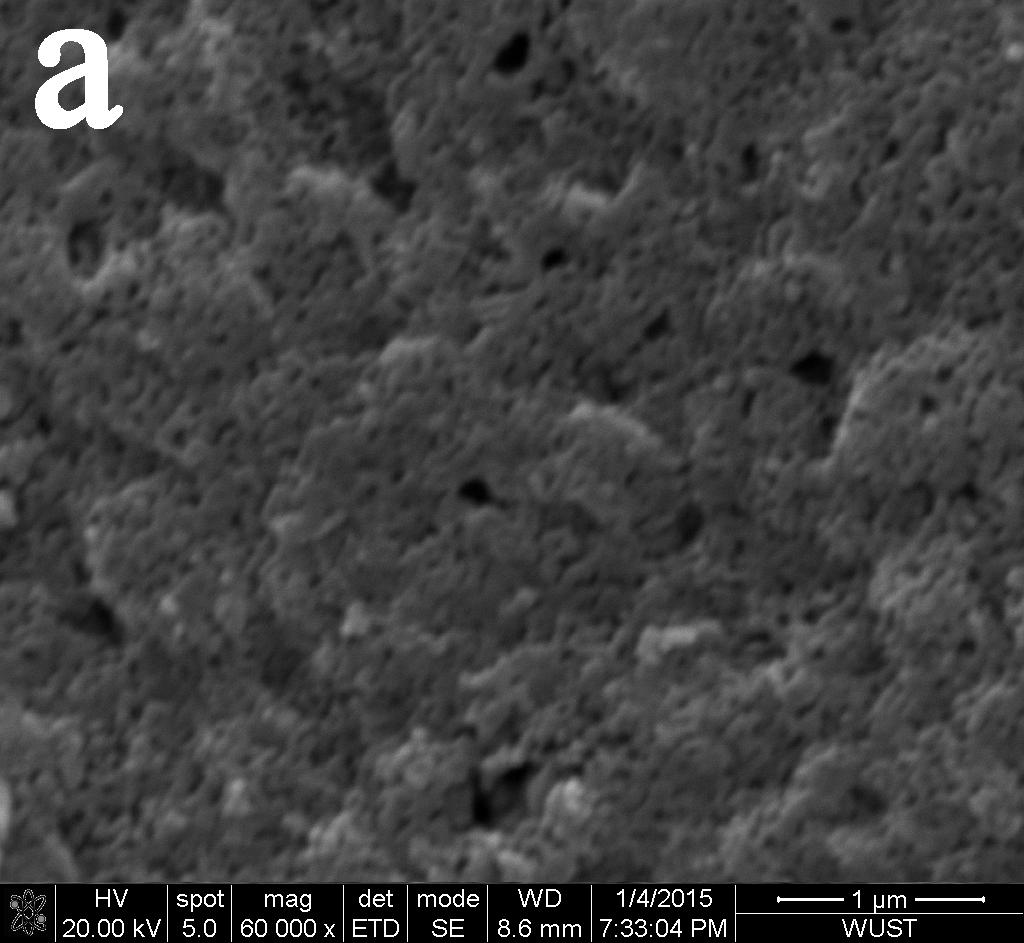

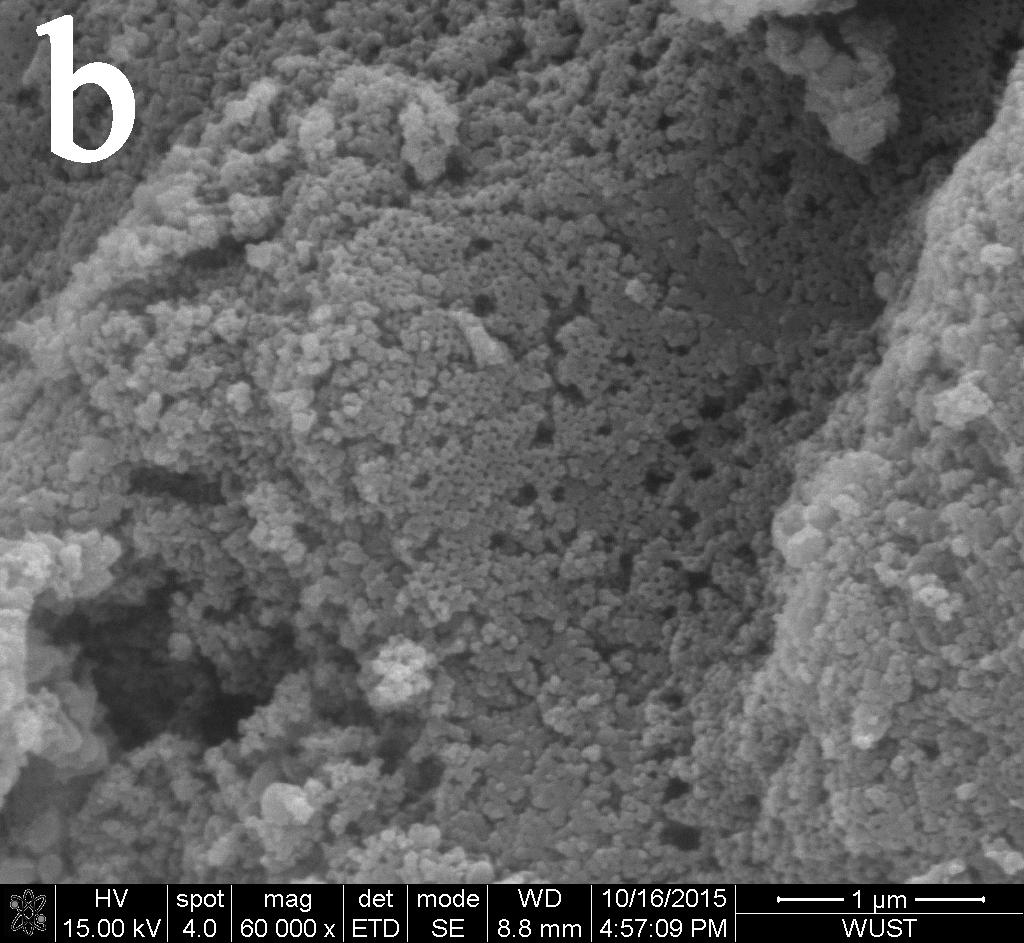

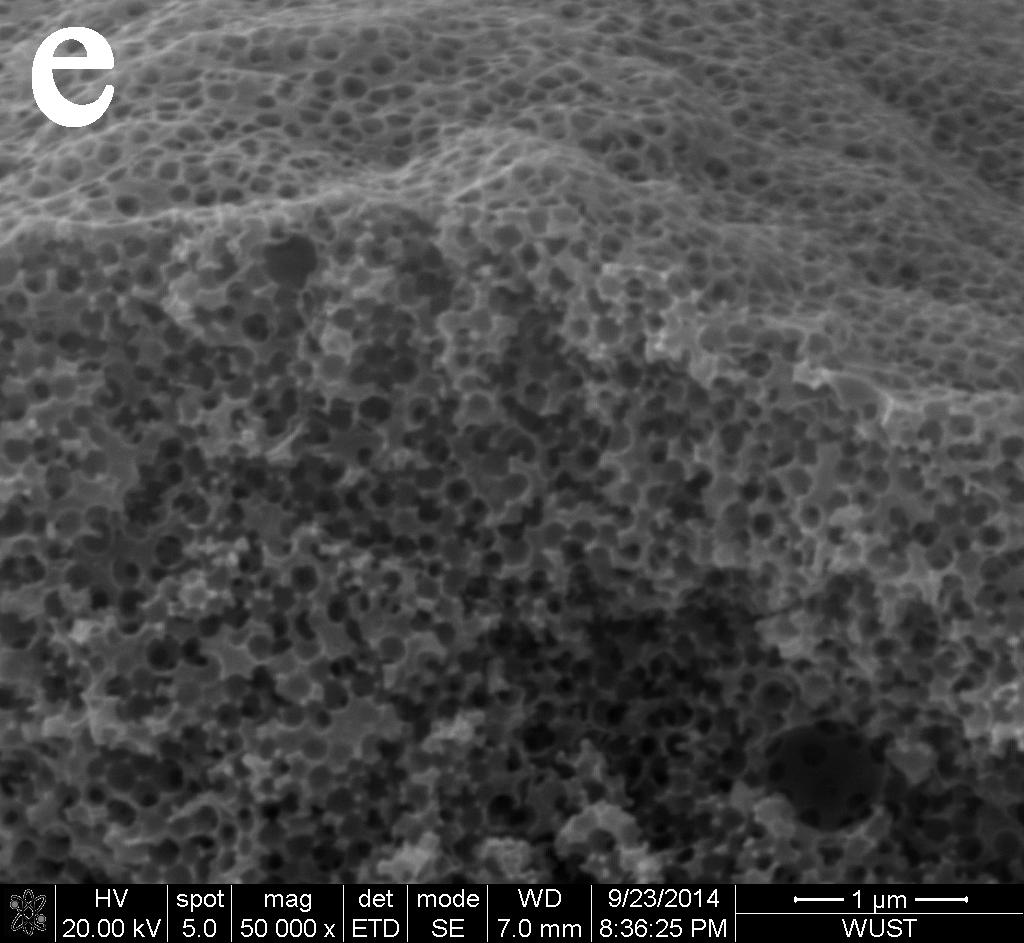

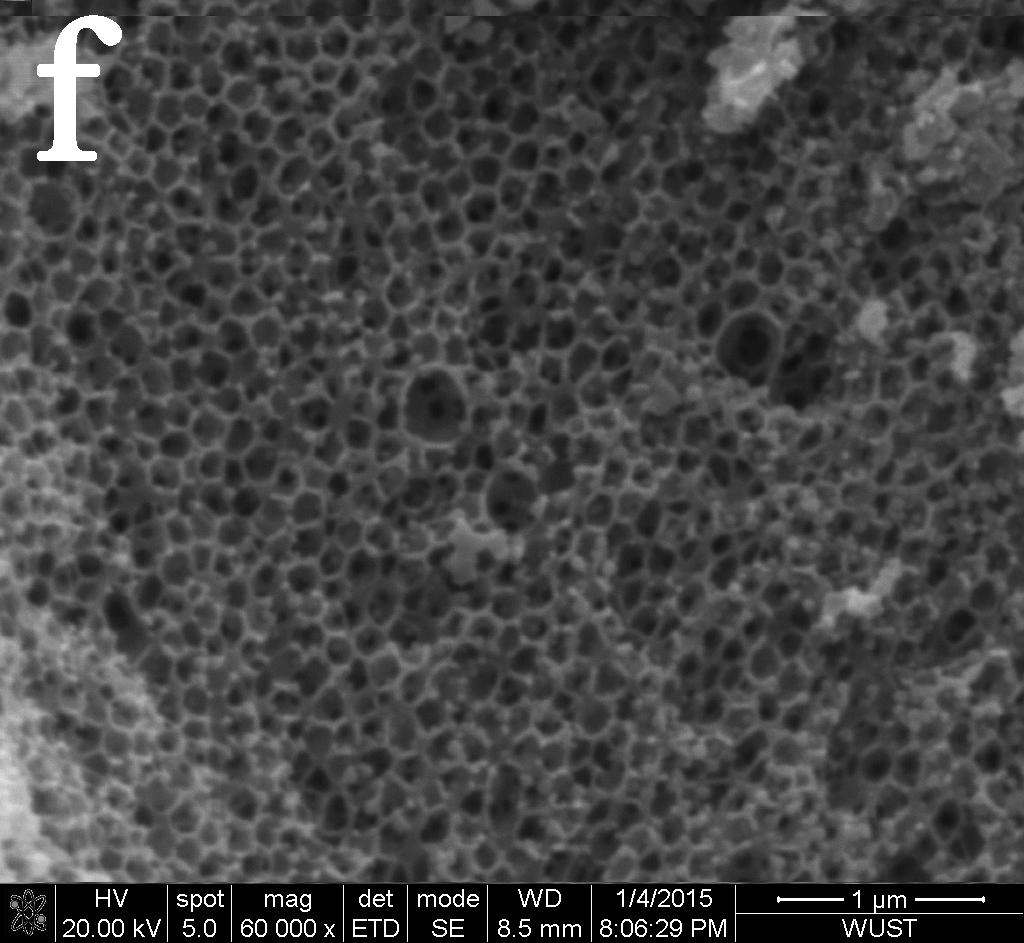

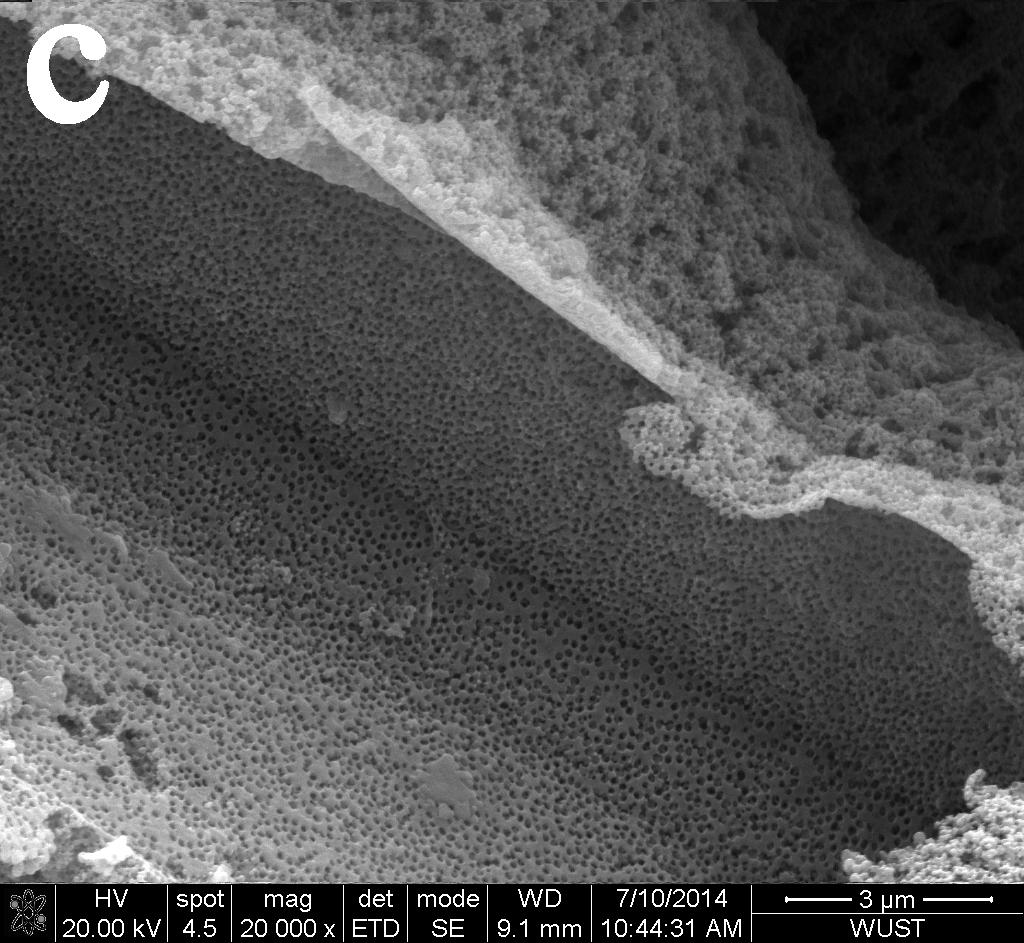

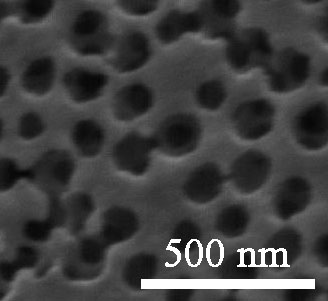

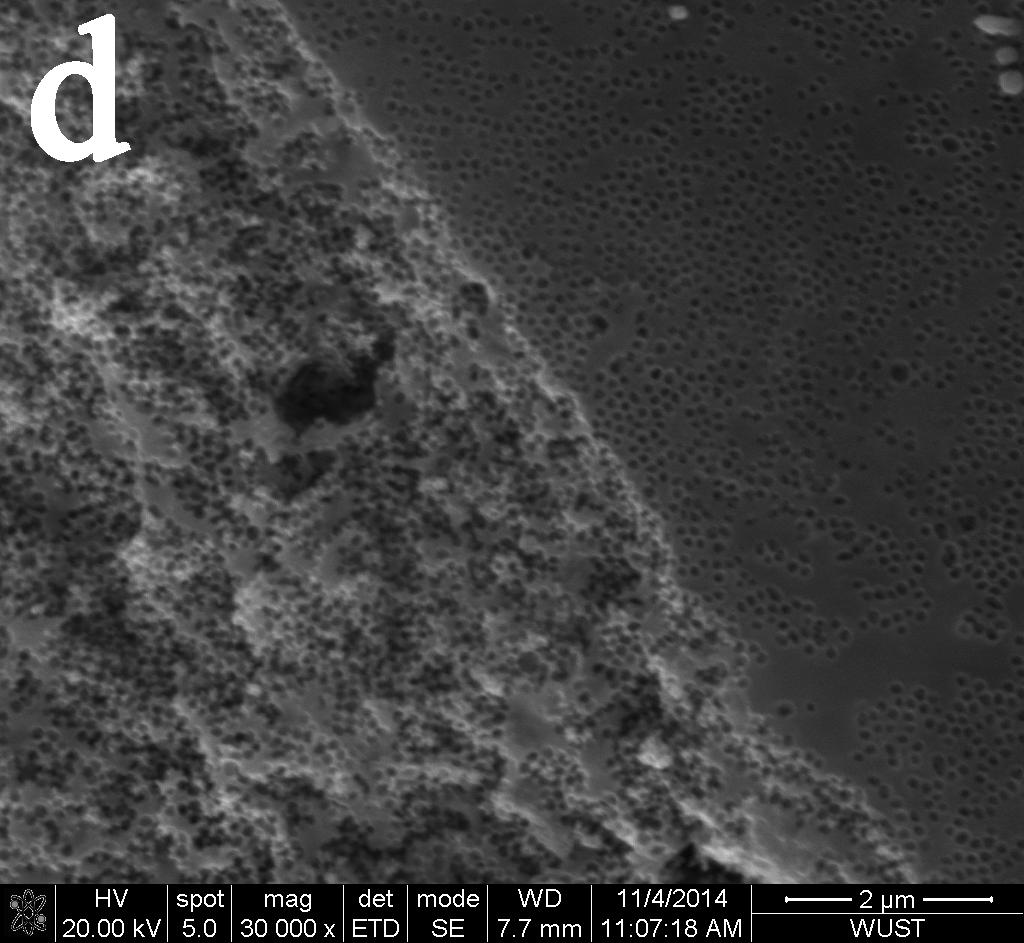

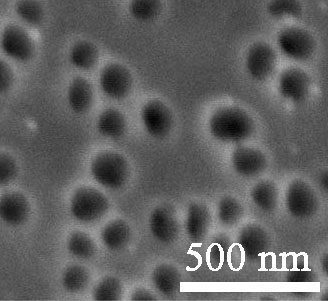


**Figure S4. SEM images of porous LiFePO_4_ materials synthesized with PMMA templates of different particle diameters.** (**a**) 30 nm; (**b**) 50 nm; (**c**) 100 nm; (**d**) 130 nm; (**e**) 180 nm; (**f**) 200 nm. For all samples, the pore sizes are in accordance with the diameters of the applied PMMA templates.

**Figure S5. XRD patterns of porous LiFePO_4_ by using PMMA templates with different particle diameters.** (**a**) 200 nm; (**b**) 130 nm; (**c**) 100 nm; (**d**) 30 nm. It shows that the diffraction peaks of all materials can be indexed to the orthorhombic LiFePO_4_, indicating that pure phase porous materials are successfully prepared.


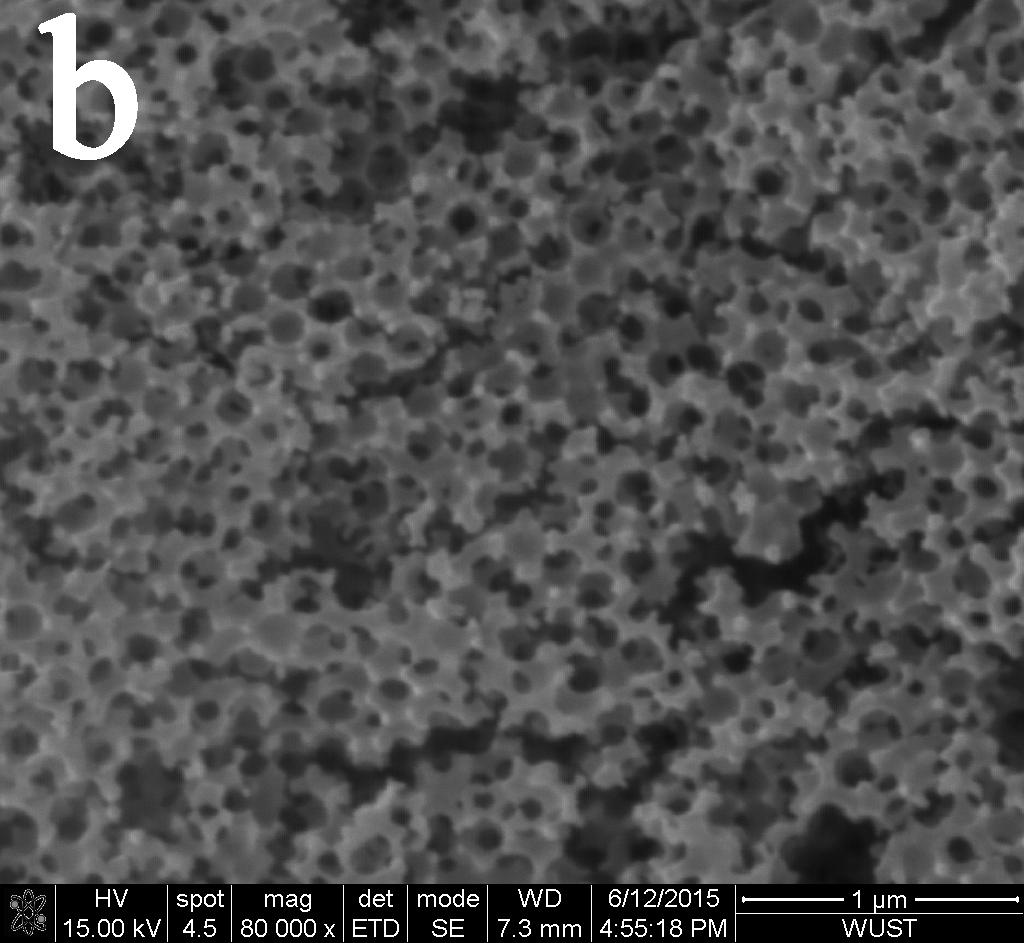

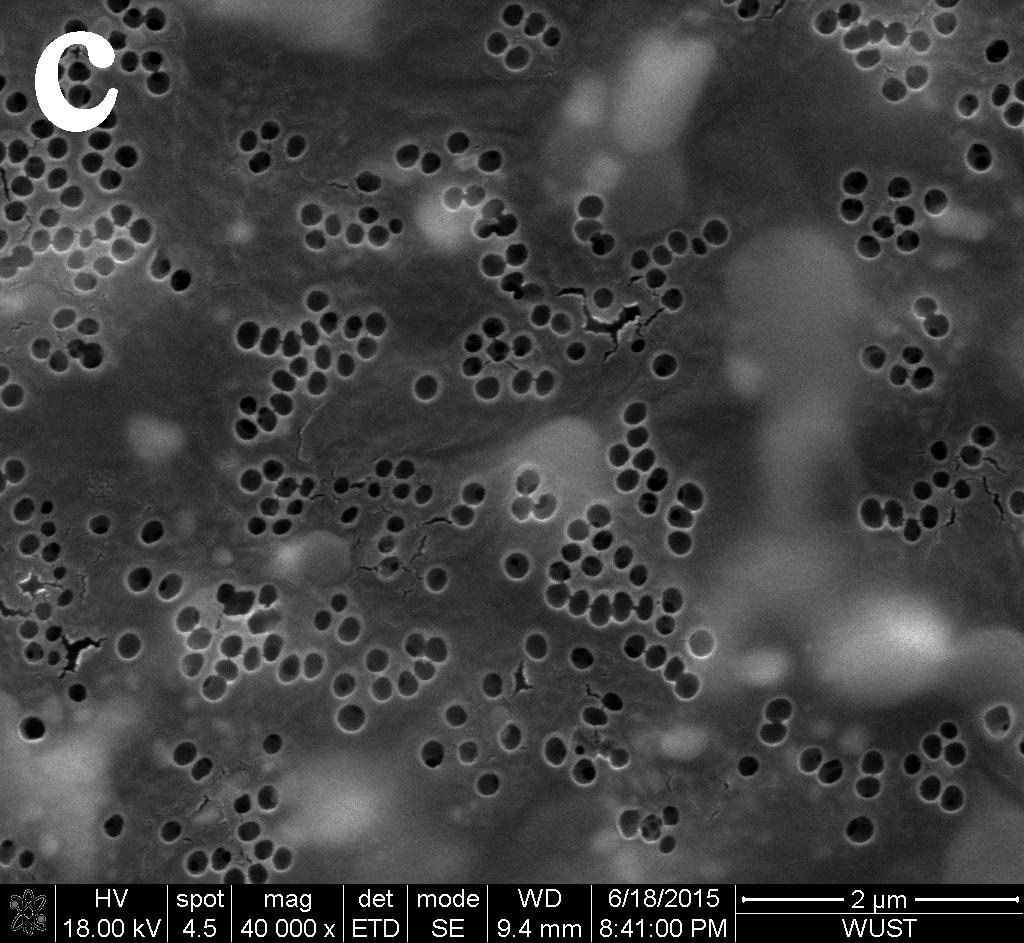

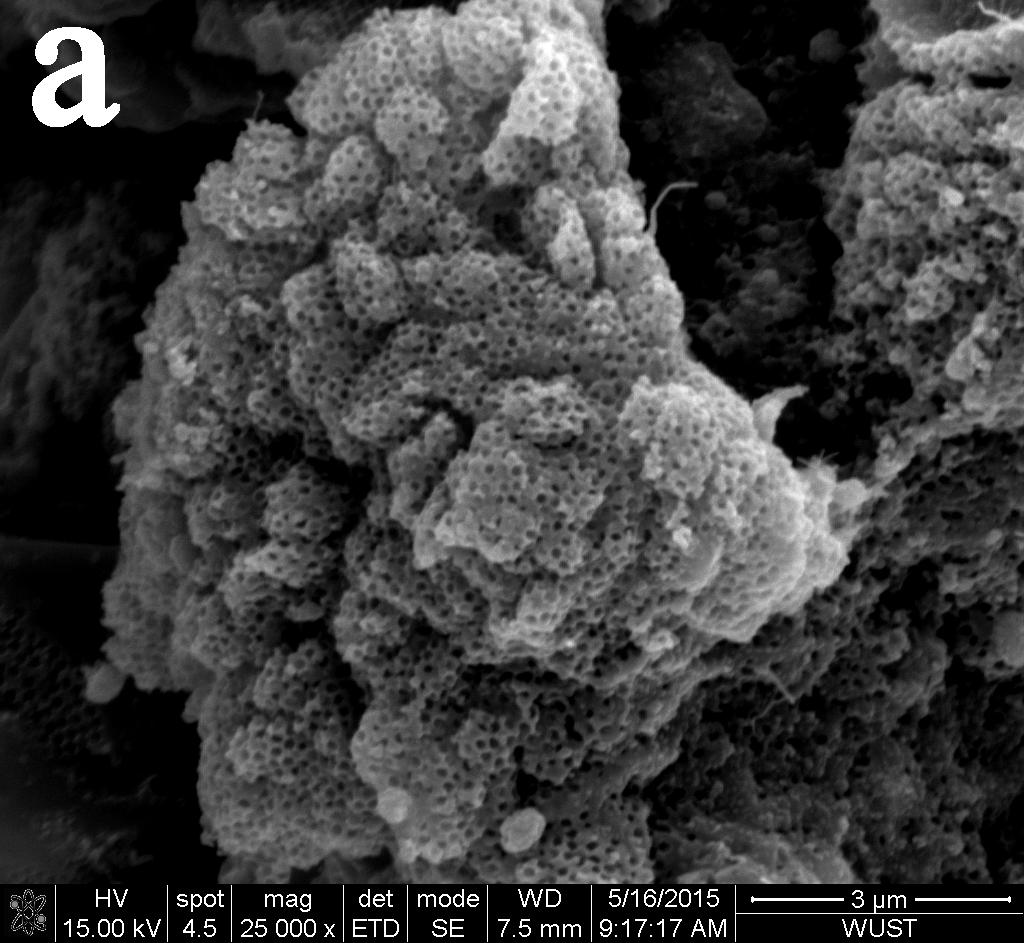

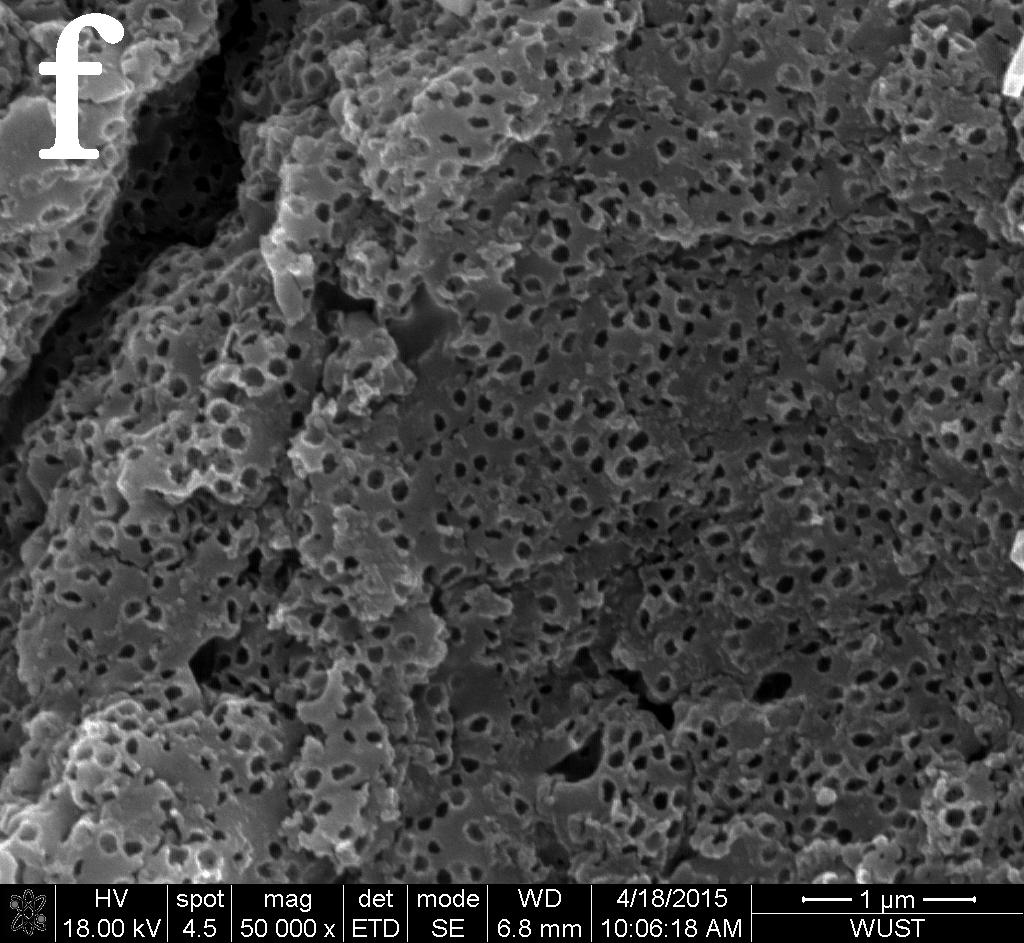

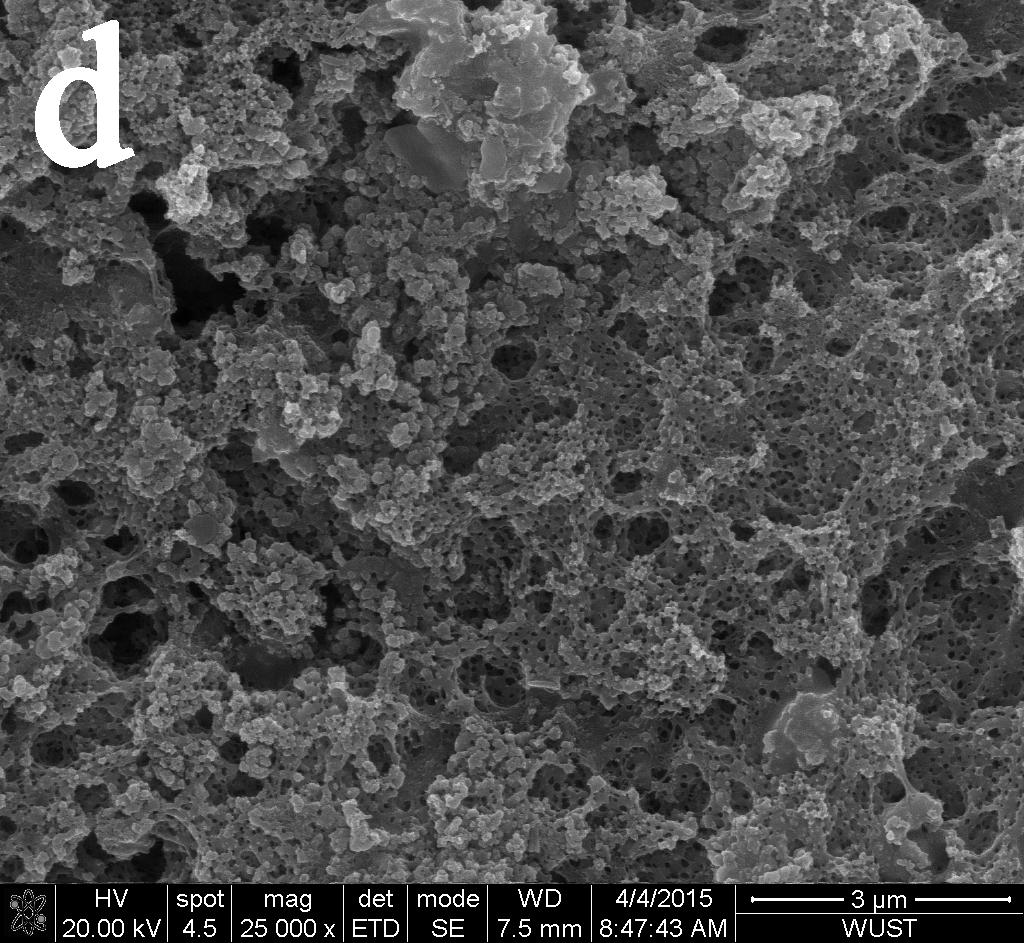

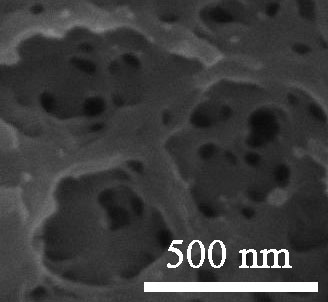

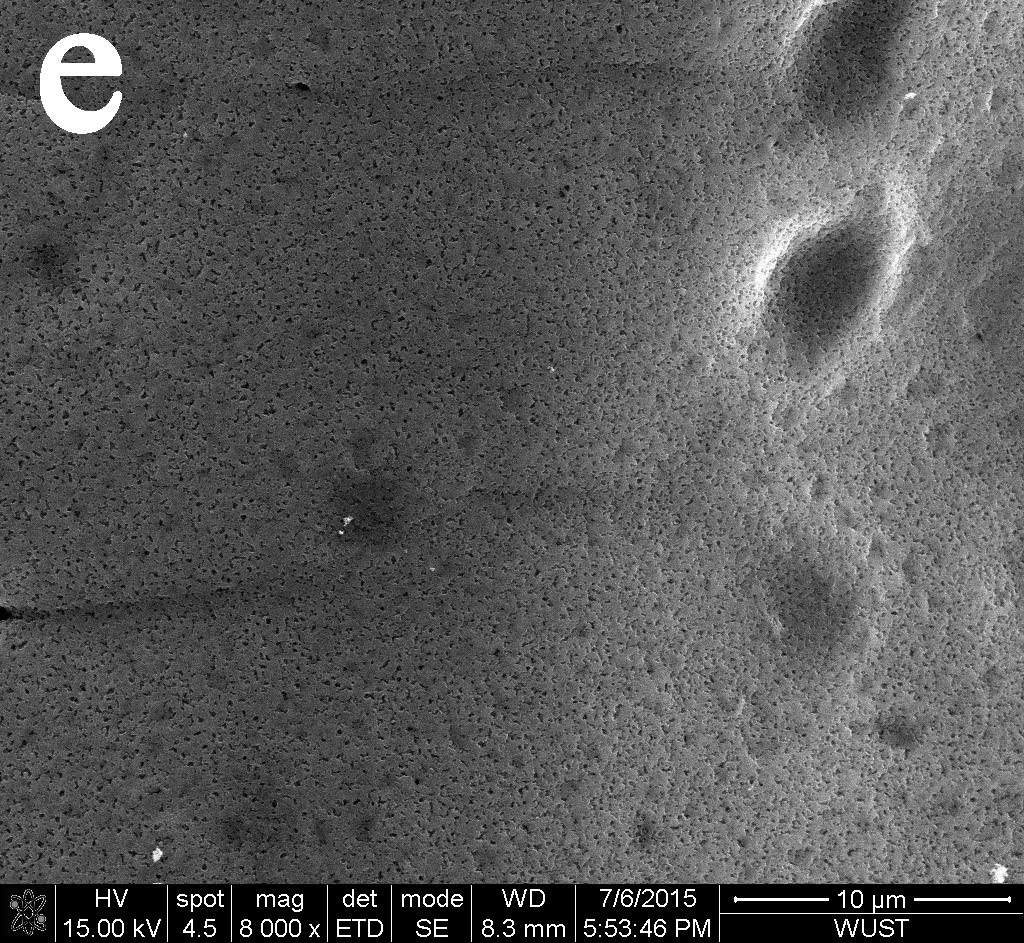

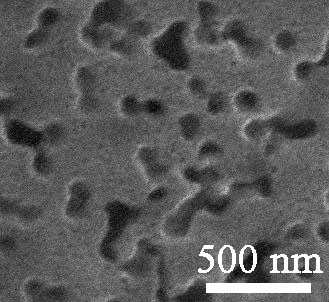


**Figure S6. SEM images of different porous materials synthesized with PMMA templates. (a**) LiMnPO_4_ synthesized with 67 wt.% PMMA of 100 nm. (**b**) LiCoPO_4_ synthesized with 67 wt.% PMMA of 100 nm. (**c**) LiNiPO_4_ synthesized with 25 wt.% PMMA of 150 nm. (**d**) LiFe_0.5_Mn_0.5_PO_4_ synthesized with 50 wt.% PMMA of 100 nm. (**e**) TiO_2_ synthesized with 67 wt.% PMMA of 150 nm. (**f**) Li_4_Ti_5_O_12_ synthesized with 50 wt.% PMMA of 100 nm.

**Figure S7. XRD patterns of various porous phosphate materials by the “pearl milk tea” freeze drying method with PMMA templates.** (**a**) LiMnPO_4_ synthesized with 67 wt.% PMMA of 100 nm; (**b**) LiCoPO_4_ synthesized with 67 wt.% PMMA of 100 nm; (**c**) LiNiPO_4_ synthesized with 25 wt.% PMMA of 150 nm; (**d**) LiFe_0.5_Mn_0.5_PO_4_ synthesized with 50 wt.% PMMA of 100 nm;

**Figure S8. XRD patterns of porous materials synthesized by the “pearl milk tea” freeze drying method with different templates.** (**a**) TiO_2_ synthesized with 67 wt.% PMMA template of 150 nm; (**b**) Li_4_Ti_5_O_12_ synthesized with 50 wt.% PMMA template of 100 nm; (**c**) LiFePO_4_ synthesized with 50 wt.% PPy template of 100 nm; (**d**) MnO_2_ synthesized with 50 wt.% SiO_2_ template of 400 nm.

**Table S1. Summary of synthesis conditions and particle diameters of PMMA templates.**

| **Sample** | **Mass of SDS (mg)** | **Volume of MMA (mL)** | **Mass of KPS (mg)** | **Reaction temperature (°C)** | **Particle diameter (nm)** |
| --- | --- | --- | --- | --- | --- |
| A | 400 | 10 | 467.6 | 70 | 30 |
| B | 200 | 15 | 116.9 | 70 | 50 |
| C | 12 | 15 | 116.9 | 70 | 100 |
| D | 5 | 15 | 29.2 | 70 | 130 |
| E | 0.6 | 15 | 29.2 | 70 | 180 |
| F | 1.05 | 15 | 1.85 | 70 | 200 |

1. ^^ Corresponding author. Tel.: +86 2768 862928, Fax: +86 2768 862928. E-mail: [zhouyk888@hotmail.com](mailto:zhouyk888@hotmail.com). [↑](#footnote-ref-2)
